# Recognizing Privilege as a Social Determinant of Health During COVID-19

**DOI:** 10.1089/heq.2020.0038

**Published:** 2020-08-27

**Authors:** Elizabeth A. Brown, Brandi M. White

**Affiliations:** ^1^ Department of Health Professions, College of Health Professions, Medical University of South Carolina (MUSC), Charleston, South Carolina, USA.; ^2^ Department of Health and Clinical Sciences, College of Health Sciences, University of Kentucky, Lexington, Kentucky, USA.

**Keywords:** social determinants of health, privilege, minority health, public health, health inequities

## Abstract

There is a need for health professionals to identify social determinants of health (SDOH) and understand the role they play in patients' health. In fall 2019, authors taught health care studies students about SDOH using a modified privilege walk to show how privilege as SDOH can impact health. This novel approach to teach about privilege and SDOH is even more relevant in today's COVID-19 world. It is our hope that those with more privilege will (1) have increased awareness and sensitivity and (2) use their voice and power to advocate for underserved communities who are disproportionately affected by historical injustices.

## Introduction

This past fall, my colleague and I modified a course to teach undergraduate students about social determinants of health (SDOH) and privilege.^1^ SDOH are “the conditions in which people are born, grow, work, live, and age, and the wider set of forces and systems shaping the conditions of daily life.”^2^ One course assignment in which students had a strong reaction to was a modified privilege walk (MPW).^3^ In general, a privilege walk will have students stand on a line, and then step forward or backward based on their responses to questions.^4^

**FIG. 1.**
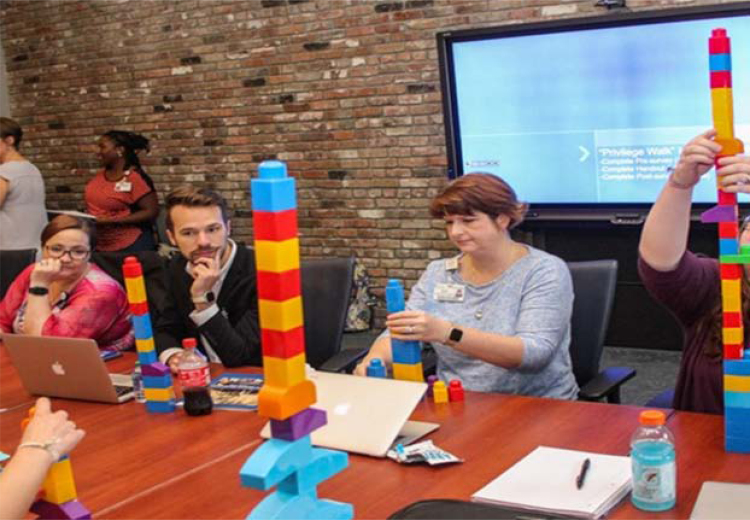
MPW. This figure portrays health care studies students building a tower based on their privilege score from a MPW in their fall 2019 social determinants of health course. During the MPW, students completed a handout related to race/ethnicity and socioeconomic status. Students then calculated a “privilege score” and used that score to get Legos and build a tower. Some students had a negative score or zero, so those students did not receive any Legos. MPW, modified privilege walk. Photo printed with permission.

In our MPW, students answered 36 questions related to race/ethnicity and socioeconomic status,^4^ counted their number of positive responses and negative responses, and calculated their “privilege score” by subtracting the number of negative responses from the positive responses. Once students calculated their score, they were given that number of Legos to build a tower going up to illustrate privilege (Fig. 1). If students had a negative score or zero, they were not given any Legos. Throughout the course, the MPW assignment encouraged discussions about the role of privilege in their lives and how it can affect others, specifically their patients, who may be marginalized or less advantaged than themselves.

Privilege as SDOH is not a new concept.^1^ For simplicity, we use Merriam-Webster's definition of privilege as a right, benefit, advantage, or opportunity.^5^ Privilege as SDOH is a meaningful concept because privilege can positively impact all SDOH. This concept can dismantle negative connotations of privilege, increase awareness of privilege, and promote those with more privilege to help the less privileged. When discussing privilege as SDOH in their final reflective article, one student wrote, “SDOH are man-made phenomena that are developed by society's perception of the value of human life. SDOH only influences health because society allows it to.” Here, with this quote, is where we can discuss the intersection of privilege, SDOH, and COVID-19.

With COVID-19, privilege has emerged (or remains) an even more powerful SDOH because of its implications on one's ability to protect themselves from COVID-19 transmission and their ability to financially support their family during a public health crisis. While working on this SDOH course project, my colleague, nor our students, would have ever imagined the application of privilege as SDOH during a pandemic.

## Historical Inequity

This pandemic has shaken the world to its core and will change life as we know it. In the United States, COVID-19 has unveiled our dirty little secret—that long-standing historical, racial, and social inequities continue to persist despite extensive public health and clinical efforts. Although COVID-19 is the culprit killing thousands of Americans, the real culprits are individuals, ideologies, and systems that create and support unfavorable SDOH (e.g., inadequate access to health care, racial inequities, inadequate access to healthy food options, and poor education systems) that continue to plague minorities, who tend to have less privilege and limited access to opportunities.

These poor SDOH disproportionately affect African Americans, Hispanics, and low-income populations, leading them to carry much of the burden of chronic conditions (diabetes, hypertension, etc.) that put them at a higher risk for severe COVID-19 outcomes, including hospitalizations and death. Unfortunately, but unsurprisingly, when we conducted our MPW with our students, on average, black students had a lower total “privilege score” than their white counterparts (−1 points vs. 18.2 points). Students who fell into other racial/ethnic minority groups, on average, scored 0.4 points. In this exercise, we saw inequity in privilege associated with race/ethnicity and socioeconomic status. We have no doubt these same privilege inequities are mirrored in American society and may be exacerbated in COVID-19 America.

We must embrace profound decisions that address these SDOH while addressing the health and well-being of Americans during the COVID-19 pandemic and moving forward. For example, policy makers should consider expanding Medicaid for those who lost their job, particularly childless adults, offering longer moratorium on evictions due to nonpayment, and promote hybrid and online learning for school-aged children. Essential workers, especially those in vulnerable populations, need more opportunities to recover; they do not have the privilege of working from home. Individuals need protected time (annual and sick leave) to care for children, elderly parents, and themselves or take time to get tested. Individuals and families need health insurance to afford COVID-19 tests and hospitalization costs.

If we use the World Health Organization's (WHO) Commission on Social Determinants of Health (CSDH) conceptual framework^6^ to promote health equity, we can already identify local, state, and federal policies that aim to keep Americans healthy. The WHO CSDH framework identifies factors or SDOH (policies, cultural values, socioeconomic status, psychosocial factors, etc.) that impact an individual's health, well-being, and quality of life. By acknowledging these factors and better understanding them, we can pinpoint policy areas to improve (e.g., demanding health insurance and protected sick time for all Americans) and promote health equity.

“Stay at Home” orders promote equity because many employers may have been legally required to shut down, providing employees the privilege or opportunity to stay home, thus avoiding COVID-19 transmission. Stimulus checks promoted equity because Americans were given financial assistance to pay bills, buy groceries, cover COVID-19 screening costs, or purchase protective equipment (masks and gloves). However, because of longstanding inequities, even these policies are not enough to help essential workers, especially those who are racial/ethnic minorities, considered low-income groups, or underprivileged.

In response to COVID-19, the federal government issued coronavirus guidelines urging Americans to stay home and contact their medical provider if they feel sick. While these guidelines may seem simple at first glance, we must consider Americans who (1) work in industries with minimal pay, (2) work jobs with no sick leave, and (3) are uninsured or underinsured. In 2018, the United States had ∼27.5 million Americans who were uninsured.^7^ Approximately 5.4% non-Hispanic whites were uninsured compared with 27.5% uninsured African Americans and Hispanics.^7^ Although the federal government issued guidelines to promote health and well-being, they have disregarded the millions of Americans, particularly minoritized racial/ethnic groups, who do not have health insurance and cannot afford adequate access to care related to COVID-19.

Ignoring the impact that poor SDOH have on the well-being of groups such as African Americans and Hispanics is not only detrimental to our public health efforts to tackle COVID-19, but it also promotes the spread of COVID-19. Unfortunately, individuals who have poor access to care, who are uninsured, or have a lower socioeconomic status may delay care and getting screened for COVID-19. Even worse is the notion that many minorities, including undocumented immigrants, are sometimes fearful and do not trust the health care system for various reasons. Lastly, essential workers who need their jobs and even childcare—those who do not have the privilege of staying home—may help spread COVID-19.

African Americans are disproportionately employed in essential positions that place them at a heightened risk of acquiring COVID-19. African Americans make up 37% of certified nursing assistants and home health aides, 34% correctional officers and jailers, and 27% of bus drivers.^8^ These employees are considered essential workers with high contact with the public, placing them at a higher risk for COVID-19 and preventable hospitalizations and deaths.

When we think about the economic impact of COVID-19 on unemployment, African Americans make up 31% of barbers,^8^ an occupation that has ceased because it is “unessential.” For barbers who cannot afford to shut their doors, they are cutting and styling hair in their homes or customers' homes, thereby increasing not only their own risk for COVID-19 exposure but also their families and customers. Racial disparities in education and job opportunities are just one result of the historical and current policies of exclusion and racism that have caused African Americans to bear a disproportionate burden of poor SDOH and now COVID-19 transmission.

## Conclusion

Unfortunately, until we, America, acknowledge and address the structural inequities that place minoritized racial/ethnic and low-income groups at a higher risk for poor health outcomes, we will be ill equipped to protect our most vulnerable populations. The COVID-19 pandemic exposes the limitations of the U.S. health care system and illustrates the disparities in SDOH that have a significant impact on health in this country.

Moving forward, every American should demand (1) adequate health and dental insurance coverage, (2) equal access to high-quality K-12 education and free college education, and (3) an acceptable amount of annual paid sick leave for every American, regardless of race/ethnicity or job title. These are the type of systemic changes we need to begin to close the gap in health care and address deep-rooted racial disparities in this country. Acknowledging our privilege or lack thereof during this pandemic is essential as our new world beyond COVID-19 continues to unfold. Society has the opportunity to change the way service and care is delivered to make sure the most vulnerable are protected—acknowledge the role of SDOH and how they impact blacks, Hispanics, and low-income groups and advocate for those groups.

## Abbreviations Used

CSDH: Commission on Social Determinants of Health
MPW: modified privilege walk
SDOH: social determinants of health
WHO: World Health Organization
